# New setting of neurally adjusted ventilatory assist for noninvasive ventilation by facial mask: a physiologic study

**DOI:** 10.1186/s13054-017-1761-7

**Published:** 2017-07-07

**Authors:** Federico Longhini, Chun Pan, Jianfeng Xie, Gianmaria Cammarota, Andrea Bruni, Eugenio Garofalo, Yi Yang, Paolo Navalesi, Haibo Qiu

**Affiliations:** 1 Anesthesia and Intensive Care, Sant’Andrea Hospital, ASL VC, Vercelli, Italy; 2 Department of Critical Care Medicine, Nanjing Zhong-Da Hospital, Southeast University School of Medicine, 87 Dingjiaqiao Road, Nanjing, 210009 China; 3 Anesthesia and Intensive Care, “Maggiore della Carità” Hospital, Novara, Italy; 4 Intensive Care Unit, University Hospital Mater Domini, Department of Medical and Surgical Sciences, Magna Graecia University, Catanzaro, Italy

**Keywords:** Noninvasive ventilation, Pressure support ventilation, Neurally adjusted ventilatory assist, Patient-ventilator interaction, Ventilator performance, Patient-ventilator asynchrony

## Abstract

**Background:**

Noninvasive ventilation (NIV) is generally delivered using pneumatically-triggered and cycled-off pressure support (PS_P_) through a mask. Neurally adjusted ventilatory assist (NAVA) is the only ventilatory mode that uses a non-pneumatic signal, i.e., diaphragm electrical activity (EAdi), to trigger and drive ventilator assistance. A specific setting to generate neurally controlled pressure support (PS_N_) was recently proposed for delivering NIV by helmet. We compared PS_N_ with PS_P_ and NAVA during NIV using a facial mask, with respect to patient comfort, gas exchange, and patient-ventilator interaction and synchrony.

**Methods:**

Three 30-minute trials of NIV were randomly delivered to 14 patients immediately after extubation to prevent post-extubation respiratory failure: (1) PS_P_, with an inspiratory support ≥8 cmH_2_O; (2) NAVA, adjusting the NAVA level to achieve a comparable peak EAdi (EAdi_peak_) as during PS_P_; and (3) PS_N_, setting the NAVA level at 15 cmH_2_O/μV with an upper airway pressure (Paw) limit to obtain the same overall Paw applied during PS_P_. We assessed patient comfort, peak inspiratory flow (PIF), time to reach PIF (PIF_time_), EAdi_peak_, arterial blood gases, pressure-time product of the first 300 ms (PTP_300-index_) and 500 ms (PTP_500-index_) after initiation of patient effort, inspiratory trigger delay (Delay_TR-insp_), and rate of asynchrony, determined as asynchrony index (AI%). The categorical variables were compared using the McNemar test, and continuous variables by the Friedman test followed by the Wilcoxon test with Bonferroni correction for multiple comparisons (*p* < 0.017).

**Results:**

PS_N_ significantly improved patient comfort, compared to both PS_P_ (*p* = 0.001) and NAVA (*p* = 0.002), without differences between the two latter (*p* = 0.08). PIF (*p* = 0.109), EAdi_peak_ (*p* = 0.931) and gas exchange were similar between modes. Compared to PS_P_ and NAVA, PS_N_ reduced PIF_time_ (*p* < 0.001), and increased PTP_300-index_ (*p* = 0.004) and PTP_500-index_ (*p* = 0.001). NAVA and PS_N_ significantly reduced Delay_TR-insp_, as opposed to PS_P_ (*p* < 0.001). During both NAVA and PS_N_, AI% was <10% in all patients, while AI% was ≥10% in 7 patients (50%) with PS_P_ (*p* = 0.023 compared with both NAVA and PS_N_).

**Conclusions:**

Compared to both PS_P_ and NAVA, PS_N_ improved comfort and patient-ventilator interaction during NIV by facial mask. PS_N_ also improved synchrony, as opposed to PS_P_ only.

**Trial registration:**

ClinicalTrials.gov, NCT03041402. Registered (retrospectively) on 2 February 2017.

## Background

Noninvasive ventilation (NIV) is increasingly used for treating acute respiratory failure (ARF) [1, 2] and is commonly applied using a facial mask [3] and pneumatically triggered and cycled-off pressure support (PS_P_) [4]. Although better tolerated than invasive mechanical ventilation, NIV is characterized by drawbacks such as poor patient-ventilator interaction and discomfort [5], which are major determinants of NIV failure.

In particular, the pneumatic signals, i.e., flow, volume and airway pressure (Paw), are leak-sensitive [6] and frequently cause patient-ventilator asynchrony [7]. The only mode not utilizing pneumatic signals to trigger and drive the ventilator is neurally adjusted ventilator assist (NAVA). In fact, with NAVA the ventilator assistance is under the control of the diaphragm electrical activity (EAdi) [8]. In contrast to PS_P_, NAVA has been repeatedly shown to improve patient-ventilator interaction and reduce asynchronies, both during invasive ventilation [9, 10] and NIV [4, 11–15]. However, NAVA is characterized by a lower rate of pressurization than PS_P_ [4].

Recently, a specific NAVA setting has been proposed to generate EAdi-controlled pressure support (PS_N_) in patients receiving either invasive ventilation [16] or NIV by helmet [4]. PS_N_ consists of increasing the user-controlled gain factor (NAVA level) at the maximum level, while limiting peak airway pressure (Paw_peak_) by adjusting the upper pressure limit [4, 16].

During NIV delivered by helmet, compared to both PS_P_ and NAVA, PS_N_ results in better pressurization and triggering performance, which improves patient comfort while reducing EAdi, without affecting the respiratory rate and gas exchange [4]. Due to the different characteristics of helmets and masks, it is unclear whether these advantages could be extended to NIV delivered by mask. This physiological study aims at comparing PS_N_ with PS_P_ and NAVA, with respect to the patient’s comfort (primary endpoint), breathing pattern, respiratory drive, gas exchange, pressurization and triggering performance and patient-ventilator synchrony (additional endpoints).

## Methods

The present physiologic, crossover, randomized study was conducted from March to September 2013 in the Intensive Care Units (ICUs) of the University Hospital “Maggiore della Carità” (Novara, Italy) and the ZhongDa Hospital, Southeast University (Nanjing, China). The study was approved by the local Ethics Committees “A.O.U Maggiore della Carità” in Novara, Italy (protocol n° 64/12) and the Research Ethics Board of Zhongda Hospital, Southeast University, Nanjing, China (2013ZDSYLL097.0). Written informed consent was obtained from the patients for publication of their individual details and accompanying images in this manuscript. The consent forms are held by the authors and are available for review by the Editor-in-Chief. At the time the study was conducted, trial registration was not mandatory for this type of investigation; however, the trial was retrospectively registered at ClinicalTrials.gov (NCT03041402). We followed the Consolidated Standards of Reporting Trials (CONSORT) recommendations for reporting of randomized trials [17].

### Patients

We considered any patient eligible who was ≥18 years of age and admitted to the ICU, and who was orally intubated and undergoing invasive mechanical ventilation for at least 48 hours. The inclusion criteria were: (1) consciousness, as indicated by a Glasgow Coma Scale (GCS) of 11 (i.e. spontaneous eye opening, response to command and no verbal response because of the endotracheal tube in place); (2) no infusion of midazolam or propofol in the previous 24 hours or 4 hours, respectively; and (3) readiness for extubation with indication, prior to extubation, to receive NIV to prevent post-extubation respiratory failure. The patients were considered to be eligible for the spontaneous breathing trial if they met the following criteria [18]: (1) GCS ≥8; (2) presence of clearly audible cough during suctioning; (3) tracheal suctioning ≤2/hour; (4) normal sodium blood values; (5) core temperature <38.5 °C during the previous 8 hours; (6) arterial oxygen tension (PaO_2_) to fraction of inspired oxygen (FiO_2_) ratio (PaO_2_/FIO_2_) ≥200 with positive end-expiratory pressure (PEEP) ≤5 cmH_2_O; (7) FiO_2_ ≤ 0.4; (8) heart rate ≤125 beats/min; and (9) systolic blood pressure >90 mmHg without epinephrine or norepinephrine infusion and with dopamine infusion ≤5 mcg/kg/min. The patients considered to be at risk of extubation failure exhibited at least one of the following: (1) more than one consecutive failure of the weaning trial [19]; (2) arterial partial pressure of carbon dioxide (PaCO_2_) >45 mmHg at the end of the 30-min spontaneous breathing trial [20]; (3) chronic respiratory disorders [19]; and (4) chronic heart failure [19].

The exclusion criteria were as follows: (1) need for analgaesic or sedative drugs; (2) recent cervical spine injury; (3) obstructive sleep apnoea syndrome; (4) pregnancy; (5) contraindications to placement of a nasal-gastric feeding tube; (6) inclusion in other research protocols; and (7) lack of consent.

### Study protocol

After the patient’s enrolment in the study, the nasal-gastric feeding tube in place was replaced by the EAdi catheter (Maquet Critical Care, Solna, Sweden) [9]. The correct positioning was ascertained as previously described [9]. The study was performed using a standard Servo-I ventilator (Maquet Critical Care, Solna, Sweden) equipped with NAVA module and NIV software for air leaks. The facial mask was individually selected for each patient based on their anthropometric characteristics to minimize air leaks and optimize patient tolerance; the facial mask was selected from among three different models: FreeMotion RT041 Non Vented Full Face Mask (Fisher and Paykel, Auckland, New Zealand); Ultra Mirage FFM-NV (ResMed, San Diego, CA, USA); and PerforMax Face Mask (Philips Respironics, Murrysville, PA, USA).

Immediately after extubation, we performed a 15-min PS_P_ trial, setting the inspiratory pressure support ≥8 cmH_2_O to obtain a tidal volume of 6–8 mL · kg^-1^ of ideal body weight, with the fastest rate of pressurization and I/E cycling at 35% of peak inspiratory flow (PIF). All patients subsequently underwent three 30-min trials in random order: (1) PS_P_, with the settings obtained in the aforementioned trial; (2) NAVA, adjusting the NAVA level in order to achieve a comparable peak EAdi (EAdi_peak_) as during the PS_P_ trial, with a safety Paw upper limit of 30 cmH_2_O [4, 15]; and (3) PS_N_, setting the NAVA level at its maximum (i.e., 15 cmH_2_O/μV), and an upper Paw limit to obtain the same overall Paw applied during the PS_P_ trial [4, 16, 21]. During both NAVA and PS_N_, the trigger sensitivity was set at 0.5 μV while the default cycling-off was 70% EAdi_peak_, as fixed by the manufacturer [21]. PEEP was set by the attending physicians in a range between 5 cmH_2_O and 10 cmH_2_O, and it remained unmodified throughout the entire study period. The FiO_2_ was regulated to obtain peripheral oxygen saturation (SpO_2_) between 94% and 96%, before starting the protocol, and it remained unmodified throughout the study period.

The three modes of ventilation were applied according to a computer-generated random sequence using sealed, opaque, numbered envelopes. The envelopes were kept in the head nurse’s office in both institutions. The envelope was opened by the nurse in charge of the patient, and the prescribed sequence of modes was communicated to the investigators.

The predefined criteria for protocol interruption were as follows: (1) need for emergency re-intubation; (2) SpO_2_ < 90%; (3) acute respiratory acidosis, as defined by PaCO_2_ > 50 mmHg and pH <7.30; (3) inability to expectorate secretions; (4) hemodynamic instability (i.e., need for continuous infusion of dopamine or dobutamine >5 μg∙kg^-1^∙min^-1^, norepinephrine >0.1 μg∙kg^-1^∙min^-1^ or epinephrine or vasopressin at any dosage to maintain mean arterial blood pressure >60 mmHg); (5) life-threatening arrhythmias or electrocardiographic signs of ischaemia; or (6) loss of 2 or more points on the GCS.

### Data acquisition and analysis

Airflow, Paw and EAdi were acquired from the ventilator using an RS232 interface at a sampling rate of 100 Hz and were recorded on a computer using dedicated software (ServoTracker V. 4.0, Maquet Critical Care, Solna, Sweden). The last minute of each trial was manually analysed off-line using customized software based on Microsoft Excel, as previously described [9].

Comfort was assessed through an 11-point numeric rating scale (NRS), as previously reported [4, 22–24]. Before protocol initiation, all patients received a detailed explanation of the NRS. The patients were asked to evaluate their comfort level, indicating a number between 0 (worst possible comfort) and 10 (best possible comfort) using an ICU-adapted large-printed scale including numbers and descriptors [23]. The scores obtained were recorded without additional indications or comments [24].

Breathing pattern was assessed by determining (1) mechanical inspiratory time (TI_mec_), breath duration (TTOT_mec_) and rate of ventilator cycling (RR_mec_) from the flow tracing, and (2) the patient’s own (neural) inspiratory time (TI_neu_), breath duration (TTOT_neu_) and respiratory rate (RR_neu_) from the EAdi tracing. The mechanical (TI/TTOT_mec_) and neural (TI/TTOT_neu_) inspiratory duty cycles were also calculated [15, 25]. Air leaks were computed over one minute as the difference between inspiratory and expiratory tidal volumes times RR_mec_ and were expressed as percentage of the exhaled volume over one minute [15, 25]. Moreover, we measured Paw_peak_, peak inspiratory flow (PIF) and the time to reach PIF from the onset of the patient’s effort (PIF_time_). EAdi_peak_ was also determined as an index of respiratory drive [26]. Gas exchange was assessed at the end of each trial by sampling arterial blood from a catheter already inserted for clinical purposes.

To evaluate the pressurization performance, we computed the pressure-time product (PTP) of the first 200 ms from the onset of the ventilator pressurization (PTP_200_), and the PTP of the first 300 ms and 500 ms from the onset of the neural effort, expressed as the percentage of the area of ideal pressurization (PTP_300-index_ and PTP_500-index_, respectively) [4, 24, 27, 28]. The ideal PTP was computed considering a perfectly squared rectangle on the Paw-time tracing, with the height of the actual Paw above PEEP and the width of the time window considered (i.e., 0.3 second and 0.5 second from the onset of the inspiratory effort, assessed from the EAdi tracing, for PTP_300-index_ and PTP_500-index_, respectively) [4, 24, 27, 28]. The triggering performance was evaluated by determining the pressure drop (ΔP_trigger_) and PTP of Paw (PTP_t_) during the triggering phase [4, 24, 27, 28].

To assess patient-ventilator synchrony, we computed the inspiratory trigger delay (Delay_TR-insp_), as the time lag between the onsets of neural inspiration and ventilator support, and the expiratory trigger delay (Delay_TR-exp_), as the time lag between the fall towards baseline of EAdi and the end of ventilator support. The time during which respiratory effort and ventilator assistance were synchronous, indexed to the TI_neu_ (Time_synch_/TI_neu_), was also computed [4, 24, 27]. The asynchrony index (AI%) was calculated as the total number of asynchronies (i.e., ineffective efforts, auto-triggers and double-triggers) divided by the sum of triggered and non-triggered breaths [7]. An AI% ≥10% was considered to indicate a clinically relevant rate of asynchronies [7].

### Statistical analysis

To detect an increase in comfort of 2.5 [4], with α risk of 0.05 and β risk of 0.20, a sample of 12 patients was deemed necessary. Because this calculation was based on a pairwise comparison and we actually compared three conditions, we applied the Bonferroni correction, which reduced the α risk from 0.05 to 0.017, increasing the sample size up to 14 patients. We used non-parametric tests because of the relatively small number of patients. The data are reported as median values (25–75% interquartile), unless otherwise specified. All continuous variables were compared between modes using the Friedman test and then by the Wilcoxon test; the Bonferroni correction was applied for multiple comparisons (*p* < 0.017). We compared the categorical data using the McNemar test. The Spearman rank correlation test was used to ascertain the correlation between each individual comfort score and the corresponding PTP_300-index_, PTP_500-index_, PTP_t_, Delay_TR-insp_, PIF and PIF_time_. For these comparisons, we considered two-sided *p* values <0.05 significant. All statistical analyses were performed using the Sigmaplot v. 12.0 (Systat Software Inc., San Jose, CA, USA). No interim analysis has been planned or conducted.

## Results

**Fig. 1:**
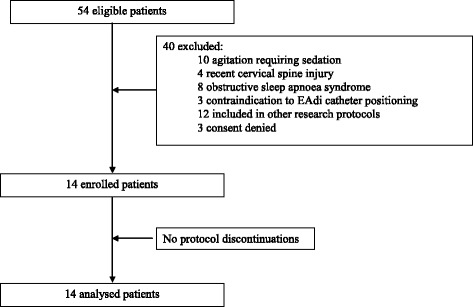
Enrolment of the study participants. The flow of patient assessment and inclusion in the protocol is shown. A total of 54 patients were considered eligible for the study, having met all inclusion criteria: 40 patients were excluded from the study because they met one or more of the exclusion criteria. Therefore, 14 patients were included in the study. No protocol discontinuations were recorded. *EAdi* diaphragm electrical activity

We enrolled 14 consecutive patients. The patients’ study flow is shown in Fig. 1. All patients completed the study protocol without any complication and were included in the data analysis. No patient required either sedative or analgaesic drugs during the study period. No patients met any criteria for post-extubation respiratory failure requiring re-intubation. The patients’ demographic and anthropometric characteristics are shown in Table 1.

**Table 1:** Patient characteristics at enrolment

Patient	Weight: kg	BMI: kg/m^2^	Admission pathology	SAPSII	PEEP: cmH_2_O	PS: cmH_2_O	FiO_2_
1	90	27.8	SE-COPD	38	10	14	0.40
2	92	29.1	SE-COPD	34	10	14	0.50
3	70	23.7	Pneumonia	28	10	10	0.40
4	87	28.2	Sepsis	37	5	15	0.35
5	75	24.5	Polytrauma	44	5	12	0.30
6	80	26.1	Polytrauma	29	5	15	0.35
7	64	23.5	Pneumonia	38	5	12	0.40
8	70	25.7	Pneumonia	38	5	8	0.50
9	60	22.0	Pneumonia	27	5	10	0.40
10	67	24.1	SE-COPD	39	7	12	0.35
11	50	19.5	Pneumonia	56	5	10	0.40
12	60	20.8	Pneumonia	57	7	12	0.40
13	58	19.6	CPE	47	8	10	0.40
14	70	25.1	Sepsis	40	5	10	0.40

*BMI* body mass index, *SAPSII* Simplified Acute Physiology Score II, *PEEP* Positive end-expiratory pressure, *PS* pressure support, *FiO*_*2*_ inspired fraction of oxygen, *SE*-*COPD* severe exacerbation of chronic obstructive pulmonary disease, *CPE* cardiac pulmonary edema

### Comfort

**Fig. 2:**
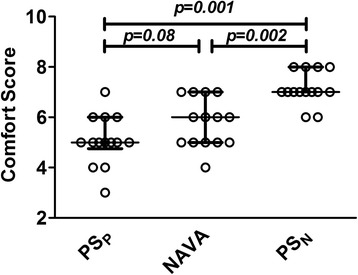
Comfort score. Individual values (*open circles*), median and interquartile range (*solid lines*) of the comfort score during pneumatically triggered pressure support (*PS*_*P*_), neurally adjusted ventilatory assist (*NAVA*) and neurally controlled pressure support (*PS*_*N*_) are depicted from *left* to *right*

The individual values of the comfort score for all the patients and their median and interquartile range are depicted in Fig. 2. PS_N_ significantly improved patient comfort (7 (7; 8)), compared to both PS_P_ (5 (5; 6); *p* = 0.001) and NAVA (5 (5; 7)); *p* = 0.002), with no differences between PS_P_ and NAVA (*p* = 0.08). Comfort was directly correlated to PTP_300-index_ (ρ =0.51, *p* < 0.001) and to PTP_500-index_ (ρ =0.46, *p*=0.002); comfort was also inversely correlated to Delay_TR-insp_ (ρ =-0.58, *p* < 0.001), PIF_time_ (ρ =-0.47, *p*=0.002) and PTPt (ρ =-0.55, *p* <0.001) while not correlated to PIF (ρ =−0.14, *p*=0.369).

### Breathing pattern, respiratory drive and gas exchange

**Fig. 3:**
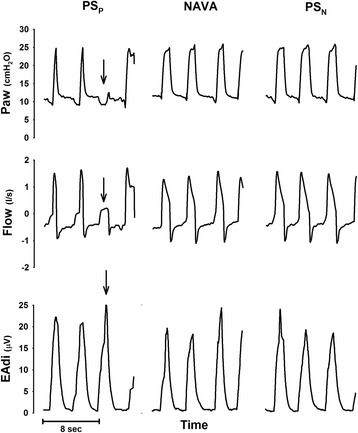
Examples of tracings from one representative patient. From *top* to *bottom*, airway pressure (*Paw*), flow and electrical activity of the diaphragm (*EAdi*) tracings of a representative patient are shown during pneumatically triggered pressure support (*PS*_*P*_), neurally adjusted ventilatory assist (*NAVA*) and neurally controlled pressure support (*PS*_*N*_). The *arrow* indicates an ineffective effort during PS_P_

As reported in Table 2, the breathing pattern was not different between modes. Only TI/TTOT_mec_ was significantly lower during PS_P_, as opposed to both NAVA (*p* = 0.007) and PS_N_ (*p* = 0.010). Paw_peak_ (*p* = 0.607), air leaks (*p* = 0.395) and respiratory drive, as indicated by the EAdi_peak_ (*p* = 0.931), were also not different between modes. PIF did not differ between the three modes of ventilation (*p* = 0.109), while PIF_time_ was significantly reduced by PS_N_, as opposed to both PS_P_ and NAVA (*p* < 0.001 for both comparison), with no differences between PS_P_ and NAVA (*p* = 0.217). Figure 3 shows, from top to bottom, Paw, flow and EAdi tracings of one representative patient undergoing PS_P_ (left), NAVA (middle) and PS_N_ (right). The arrow indicates an ineffective inspiratory effort during PS_P_. The median group values are presented in Table 2.

**Table 2:** Breathing pattern, respiratory drive, gas exchange, pressurization and triggering performance and patient-ventilator synchrony

	Friedman test (*p* value)	PS_P_	NAVA	PS_N_
*Breathing pattern and respiratory drive*				
RR_mec_ (breaths/min)	0.606	23.9 (18.7; 30.6)	26.7 (19.5; 30.6)	27.4 (18.4; 31.7)
RR_neu_ (breaths/min)	0.931	25.7 (18.6; 32.9)	26.2 (19.6; 30.7)	26.4 (19.3; 30.8)
TI_mec_ (sec)	0.168	0.71 (0.58; 0.87)	0.83 (0.61; 1.11)	0.82 (0.66; 1.04)
TI_neu_ (sec)	0.606	0.75 (0.56; 1.10)	0.74 (0.59; 1.10)	0.75 (0.59; 0.96)
TI/TTOT_mec_	0.030	0.30 (0.27; 0.33)	0.33 (0.31; 0.40)*	0.34 (0.29; 0.41)^#^
TI/TTOT_neu_	0.606	0.32 (0.26; 0.37)	0.32 (0.28; 0.38)	0.30 (0.26; 0.34)
Paw_peak_	0.607	19.3 (15.1; 21.1)	18.8 (15.4; 21.0)	19.0 (15.2; 20.5)
Leaks %	0.395	21.4 (8.9; 43.2)	35.9 (15.2; 47.6)	23.2 (11.5; 61.9)
PIF (l/sec)	0.109	1.12 (0.85; 1.42)	1.05 (0.71; 1.22)	1.20 (0.77; 1.38)
PIF_time_ (sec)	<0.001	0.41 (0.34–0.48)	0.41 (0.33–0.58)	0.22 (0.19–0.26)^#§^
EAdi_peak_ (μV)	0.257	13.7 (7.7; 21.2)	15.3 (8.4; 25.7)	12.6 (6.9; 19.3)
*Gas exchange*				
pH	0.4576	7.43 (7.40; 7.45)	7.43 (7.40; 7.45)	7.43 (7.40; 7.45)
PaCO_2_	0.5134	44.1 (36.2; 50.3)	44.4 (36.1; 51.5)	43.8 (38.2; 50.8)
PaO_2_/FiO_2_	0.5103	213.6 (197.9; 224.0)	214.6 (188.1; 238.0)	214.4 (199.0; 226.2)
*Pressurization and triggering performance*				
PTP_300-index_ (%)	0.004	24.7 (4.3; 32.7)	25.3 (19.9; 34.0)	42.0 (32.5; 46.5)^#§^
PTP_500-index_ (%)	0.001	44.2 (23.3; 52.1)	46.4 (33.4; 56.6)	62.6 (54.1; 67.9)^#§^
PTP_200_ (cmH_2_O/sec)	0.001	86.7 (77.5; 112.5)	62.1 (45.7; 81.9)*	85.0 (69.6; 127.4)^§^
PTPt (cmH_2_O/sec)	<0.001	9.45 (5.89; 12.31)	0.89 (0.23; 3.23)*	0.59 (0.16; 2.33)^#^
ΔP_trigger_ (cmH_2_O)	<0.001	−1.16 (−1.40; −0.87)	−0.36 (−0.78; −0.11)*	−0.32 (−0.71; −0.11)^#^
*Patient ventilator synchrony*				
Delay_TR-insp_ (sec)	<0.001	0.13 (0.08; 0.27)	0.07 (0.03; 0.06)*	0.05 (0.04; 0.06)^#^
Delay_TR-exp_ (sec)	0.395	0.13 (0.05; 0.22)	0.10 (0.09; 0.14)	0.11 (0.10; 0.12)
Time_synch_/TI_neu_	0.010	0.79 (0.70; 0.88)	0.90 (0.86; 0.94)*	0.94 (0.89; 0.98)^#^
AI% (%)	<0.001	6.6 (0.0; 23.4)	0.0 (0.0; 0.0)*	0.0 (0.0; 0.0)^#^

*PS*_*P*_ pneumatically triggered and cycled-off pressure support, *NAVA* neurally adjusted ventilatory assist, *PS*_*N*_ neurally controlled pressure support, *RR*_*mec*_ ventilator respiratory rate, *RR*_*neu*_ patient’s respiratory rate, *TI*_*mec*_ inspiratory time of the ventilator, *TI*_*neu*_ inspiratory time of the patient, *TI*/*TOT*_*mec*_ ventilator inspiratory duty cycle, *TI*/*TOT*_*neu*_ patient’s inspiratory duty cycle, *Paw*_*peak*_ peak airway pressure, *PIF* peak inspiratory flow, *PIF*_*time*_ time to reach the PIF, *EAdi* electrical activity of the diaphragm, *EAdi*_*peak*_ peak value of EAdi, *PaCO*_*2*_ arterial partial pressure of carbon dioxide, *PaO*_*2*_/*FiO*_*2*_ ratio between arterial partial pressure and inspired fraction of oxygen, *PTP* pressure time product, *PTP*_*300*-*index*_ PTP of the first 300 ms since the effort of the patient indexed to the ideal PTP, *PTP*_*500*-*index*_ PTP of the first 500 ms since the effort of the patient indexed to the ideal PTP, *PTP*_*200*_ PTP of the first 200 ms since the beginning of pressurization, *PTPt* PTP of the trigger, Δ*P*_*trigger*_ drop of pressure during triggering phase, *Delay*_*TR*-*insp*_ inspiratory trigger delay, *Dealy*_*TR*-*exp*_ expiratory trigger delay, *Time*_*synch*_/*TI*_*neu*_ synchronous time between respiratory effort and ventilator assistance, indexed to the TI_neu_, *AI*% asynchrony index. *p < 0.017 PS_P_ vs. NAVA, ^#^ *p* < 0.017 PS_P_ vs. PS_N_, ^§^ *p* < 0.017 NAVA vs. PS_N_

Gas exchanges were no different between trials (Table 2).

### Pressurization and triggering performance

**Fig. 4:**
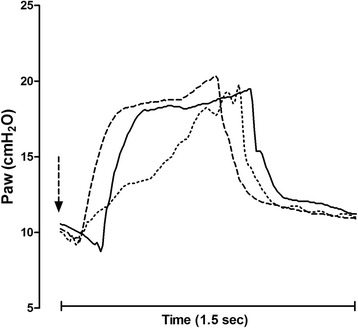
Pressure airway profiles. Airway pressure (*Paw*) profile of single breaths during pneumatically triggered pressure support (*solid line*), neurally adjusted ventilatory assist (*dotted line*) and neurally controlled pressure support (*dashed line*) from another patient. The *arrow* indicates the beginning of the patient’s effort. See main text for additional explanation

Figure 4 depicts Paw profiles of individual breaths during PS_P_ (solid line), NAVA (dotted line) and PS_N_ (dashed line) from another patient. The arrow indicates the beginning of the patient’s own (neural) effort. PS_P_ and PS_N_ have similar Paw profiles, characterized by a fast rate of pressurization; however, during PS_N_ the beginning of pressurization is notably anticipated and closer to the onset of the patient’s effort. NAVA is characterized by a slower rate of pressurization. Consistent with these findings, PS_N_ improved both PTP_300-index_ and PTP_500-index_, as opposed to both PS_P_ and NAVA (Table 2), whereas PTP_200_ was lower during NAVA, as compared to both PS_P_ and PS_N_ (*p* < 0.001 for both comparisons), with no significant difference between PS_P_ and PS_N_ (*p* = 0.761). Shown also in Table 2, NAVA and PS_N_ significantly reduced Delay_TR-insp_, PTPt and ΔP_trigger_, in contrast to PS_P_ (*p* < 0.001 for all comparisons). Delay_TR-exp_ was no different between modes (*p* = 0.395).

### Patient-ventilator synchrony

Compared to PS_P_, both NAVA (*p* = 0.005) and PS_N_ (*p* = 0.002) improved Time_synch_/TI_neu_, with no differences between the two (*p* = 0.08) (Table 2). The median values of AI% are reported in Table 2. As expected, during both NAVA and PS_N_, the AI% was <10% in all patients, whereas it was ≥10% in 7 patients (50%) with PS_P_ (*p* = 0.023, compared to both NAVA and PS_N_).

## Discussion

This physiologic study shows that in patients receiving NIV by facial mask, compared to both PS_P_ and NAVA, PS_N_ improves pressurization and triggering performance, resulting in better comfort, while not affecting respiratory drive, Arterial Blood Gases ABGs and respiratory rate. Both PS_N_ and NAVA equally improve patient-ventilator synchrony, in contrast to PS_P_.

To the best of our knowledge, this investigation is the first to evaluate PS_N_ for delivery of NIV using a mask. In a study evaluating intubated patients with COPD and intrinsic PEEP, compared to PS_P_, PS_N_ improved patient-ventilator interaction and synchrony, and counterbalanced the extra load due to intrinsic PEEP without the need for externally applied PEEP [16]. In healthy volunteers, comfort was reduced when increasing the level of support [29], whereas it was improved by EAdi triggering, as opposed to pneumatic triggering, during NIV delivered by helmet [30]. In a recent study comparing PS_N_ with PS_P_ and NAVA during NIV delivered by helmet in an analogous patient population, PS_N_ improved comfort, pressurization and triggering performance, and reduced EAdi, without affecting gas exchange [4].

Consistent with the results of these investigations, in the present study PS_N_ outperforms PS_P_ with respect to PTP_300-index_ and PTP_500-index_, PTPt [4, 16, 30], Delay_TR-insp_, Time_synch_/TI_neu_ and AI [4, 16, 30], and comfort [4, 30]. In accordance with Cammarota et al. [4], who compared the same three modes delivering NIV by helmet, PS_N_ improved pressurization PTP_300-index_ and PTP_500-index_, and comfort with respect to both PS_P_ and NAVA, while in contrast to that study, PS_N_ neither increased PTP_200_, compared to PS_P_, nor reduced EAdi, compared to both PS_P_ and NAVA. These discrepancies are likely due to the different physical properties of mask and helmet, the latter being characterized by more problematic triggering and pressurization performance [31]. Nonetheless, we found improvements in triggering and pressurization performance to ameliorate comfort, which is a major determinant of NIV outcome. Indeed, NIV can be complicated by discomfort, which is associated with increased rate of failure and worsened patient outcome [32].

PIF was not different between modes, while PIF_time_ was shortened by PS_N_, as opposed to both PS_P_ and NAVA. In intubated patients with acute on chronic respiratory failure undergoing PS_P_, Bonmarchand et al. evaluated the effects of varying Paw rates of pressurization; they found that the fastest rate generated the highest PIF and was associated with greater reduction in the work of breathing [33]. Similar results were obtained during invasive PS_N_ in restrictive patients [34] and in patients recovering from hypoxemic ARF [35].

To explain the differences between these studies and our investigation, it is important to note the different computational approach to the pressurization indexes [27]. PTP_200_ reflects the sole rate of pressurization rate, i.e., the slope of Paw after triggering, which affects the PIF. Both PTP_300-index_ and PTP_500-index_ instead consider not only the pressurization rate but also the triggering performance, which influences PIF_time_, without affecting PIF. We found PTP_200_ no different between PS_N_ and PS_P_, while triggering performance was significantly improved by PS_N_, as indicated by the lower values of PTPt and Delay_TR-insp_. Notably, while patient comfort is improved when flow delivery by the ventilator meets the patient’s demand [36], excessively high PIF may worsen the patient’s comfort during both invasive ventilation [37] and NIV [36].

Our study has two limitations. First, the patient sample is small, a limitation that we share with the majority of earlier physiological investigations [4, 9, 11–13, 15, 24, 37, 38]. Second, consistent with the results of previous research [4, 22–24], we applied the 11-point NRS to assess comfort, although this scale has been formally validated for pain [39, 40] and dyspnoea [41] only.

## Conclusions

Compared to both PS_P_ and NAVA, in patients receiving NIV by facial mask, PS_N_ improves triggering performance and patient-ventilator synchrony, thereby ameliorating the patient’s comfort. It remains to be determined whether these physiologic benefits may also occur in other categories of patients and translate into improved clinical outcomes.

## Abbreviations

AI%: Asynchrony index
ARF: Acute respiratory failure
COPD: Chronic obstructive pulmonary disease
Delay_TR-exp_: Expiratory trigger delay
Delay_TR-insp_: Inspiratory trigger delay
EAdi: Diaphragm electrical activity
EAdi_peak_: Peak of electrical activity of the diaphragm
FiO_2_: Inspiratory oxygen fraction
GCS: Glasgow Coma Scale
ICUs: Intensive Care Units
NAVA: Neurally adjusted ventilatory assist
NIV: Noninvasive ventilation
NRS: Numeric rating scale
PaCO_2_: Arterial partial pressure of carbon dioxide
Paw: Airway pressure
Paw_peak_: Peak of airway pressure
PEEP: Positive end-expiratory pressure
PIF: Peak inspiratory flow
PIF_time_: Time to reach the peak inspiratory flow from the onset of patient’s effort
PS_N_: Neurally controlled pressure support
PS_P_: Pneumatically triggered and cycled-off pressure support
PTP: Pressure-time product
PTP_200_: Pressure-time product of the first 200 ms from the onset of the ventilator pressurization
PTP_300-index_: Pressure-time product of the first 300 ms from the onset of the neural effort, indexed to the ideal area
PTP_500-index_: Pressure-time product of the first 500 ms from the onset of the neural effort, indexed to the ideal area
PTP_t_: Pressure-time product of the triggering phase
RR_mec_: Rate of ventilator cycling
RR_neu_: Patient’s own (neural) respiratory rate
SpO_2_: Peripheral oxygen saturation
TI/TTOT_mec_: Mechanical inspiratory duty cycle
TI/TTOT_neu_: Patient’s own (neural) inspiratory duty cycle
TI_mec_: Mechanical inspiratory time
Time_synch_/TI_neu_: Time during which respiratory effort and ventilator assistance are synchronous, indexed to the patient’s own (neural) inspiratory time
TI_neu_: Patient’s own (neural) inspiratory time
TTOT_mec_: Total mechanical respiratory time
TTOT_neu_: Total patient’s own (neural) respiratory time
ΔP_trigger_: Pressure drop of the triggering phase
